# Adipose mesenchymal stem cell-derived exosomes rescue mitochondrial function through SIRT1 to improve diabetic wound healing

**DOI:** 10.1093/burnst/tkaf017

**Published:** 2025-04-17

**Authors:** Xiaozhi Bai, Yu Li, Peng Wang, Zhigang Xu, Jingtao Wei, Ting He, Juntao Han

**Affiliations:** Department of Burns and Cutaneous Surgery, Xijing Hospital, Air Force Medical University, 127 Changle West Road, Xi’an, Shaanxi province 710032, China; Department of Burns and Cutaneous Surgery, Xijing Hospital, Air Force Medical University, 127 Changle West Road, Xi’an, Shaanxi province 710032, China; Department of Burns and Cutaneous Surgery, Xijing Hospital, Air Force Medical University, 127 Changle West Road, Xi’an, Shaanxi province 710032, China; Department of Burns and Cutaneous Surgery, Xijing Hospital, Air Force Medical University, 127 Changle West Road, Xi’an, Shaanxi province 710032, China; Department of Burns and Cutaneous Surgery, Xijing Hospital, Air Force Medical University, 127 Changle West Road, Xi’an, Shaanxi province 710032, China; Department of Burns and Cutaneous Surgery, Xijing Hospital, Air Force Medical University, 127 Changle West Road, Xi’an, Shaanxi province 710032, China; Department of Burns and Cutaneous Surgery, Xijing Hospital, Air Force Medical University, 127 Changle West Road, Xi’an, Shaanxi province 710032, China

**Keywords:** Diabetic wounds, Adipose mesenchymal stem cell derived-exosomes, SIRT1, Mitochondrial, Autophagy flux, Lysosome

## Abstract

**Background:**

Diabetic wounds represent the most common type of chronic wounds. Persistent inflammation and elevated oxidative stress are hallmark features of chronic wounds, where macrophage phenotypic polarization playing a critical role in the healing process. Adipose-derived mesenchymal stem cell exosomes (ADSC-exos) have shown promising therapeutic effects in the treatment of diabetic wounds by modulating macrophage function. This study aims to elucidate the specific downstream regulatory mechanisms through both *in vitro* and *in vivo* investigations.

**Methods:**

A streptozotocin-induced diabetic mouse model and high glucose-stimulated RAW 264.7 macrophages were utilized to mimic diabetic microenvironments. Wound tissues were collected from patients with diabetic foot ulcer. A skin incision model was established in mice and ADSC-exos were given subcutaneously. Streptozotocin-induced diabetic myeloid-specific *sirt1*^−/−^ mice SIRT1 siRNA-transfected macrophages were employed to investigate the role of SIRT1 *in vivo* and *in vitro*. Wound healing rates were quantified. Mitochondrial function, lysosomal activity, autophagy flux, and inflammation status were systematically assessed.

**Results:**

In diabetic mice and high glucose-treated macrophages, lysosomal dysfunction preceded mitochondrial and autophagy flux impairments. SIRT1 expression was significantly reduced in both diabetic wound tissues and macrophages, accompanied by M1 macrophage polarization. SIRT1 interference experiments revealed that the impact of ADSC-exos on mitochondrial function, autophagy flux, and inflammatory response were partially dependent on SIRT1. Notably, the therapeutic effects of ADSC-exos on mitochondrial and autophagic pathways were markedly attenuated upon SIRT1 suppression.

**Conclusions:**

These findings demonstrate that ADSC-exos promotes diabetic wound healing by restoring mitochondrial function and autophagy via SIRT1 activation. These findings highlight the therapeutic potential of ADSC-exos and provide a mechanistic foundation for future exosome engineering strategies.

## Graphical Abstract

**Graphical Abstract:**
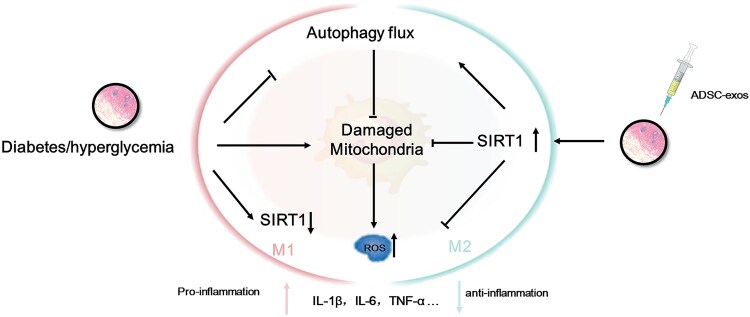

Highlights

- ADSC-exos mitigates inflammation and enhances healing in diabetic wounds.
- Diabetes impairs macrophage mitochondrial function, autophagy, and SIRT1 expression.
- Macrophage mitochondrial dysfunction disrupts lysosomal activity and autophagy, driving M1 polarization.
- ADSC-exos boosts SIRT1, restoring mitochondrial/lysosomal function and autophagy to reverse M1 polarization.
- SIRT1 enhances mitochondrial function, reshaping macrophage immune dynamics and offering therapeutic potential for diabetic wounds.

## Background

The ‘2021 IDF Global Diabetes Map (10th Edition)’ by the International Diabetes Federation (IDF) reveals that globally, there are almost 537 million adults suffering from diabetes, and about 25% of them are affected by diabetic wounds [1]. As one of the most serious complications of diabetes, JAMA even reports that the amputation rate of diabetic foot ulcers can reach 70% [2, 3], highlighting the urgent need for effective management. Macrophages play a pivotal role during wound healing by eliminating pathogens and regulating the proliferative phase of repair through paracrine signaling [4, 5]. However, the pathogenesis of chronic diabetic wounds is complex, and current research suggests that impaired macrophage polarization is a critical factor contributing to the stalled inflammatory phase and subsequent healing impairments in diabetic patients [6, 7]. Then, macrophages polarize toward the M2 phenotype to modulate tissue remodeling and suppress inflammation [8]. In both diabetic patients and mice, approximately 80% of the cells at the edges of chronic wounds are proinflammatory M1 macrophages, which do not transition promptly to the anti-inflammatory M2 phenotype [9]. These studies support the view that the ordered transformation and functional homeostasis of macrophages play crucial roles in maintaining the stability of the microenvironment in diabetic wounds. The balance of macrophage polarization is intricately linked to mitochondrial function and autophagy [10]. Mitochondria play a pivotal role in oxidative stress in wounds [11]. Within the diabetic wound environment, macrophage mitochondrial dysfunction may arise, leading to increased level of reactive oxygen species (ROS) and promoting a shift toward the M1 proinflammatory phenotype [12]. Emerging research suggests that macrophage autophagy may exert protective effects in metabolic disorders, as it clears aberrant proteins and organelles, dampens inflammasome activation, and attenuates the secretion of inflammatory cytokines, thereby preserving intracellular homeostasis [13, 14]. Impaired mitochondrial function triggers ROS generation and lysosome dysfunction, obstructing autophagic flux in macrophages [10]. Dysfunctional autophagic flux can further drive macrophage polarization toward the M1 phenotype, contributing to chronic and slight inflammation [10].

Sirtuin 1 (SIRT1), which belongs to the NAD^+^-dependent deacetylase family, functions as an NAD-dependent histone deacetylase. SIRT1 has a transcriptional regulatory function in glucose metabolism, inflammatory homeostasis, and longevity and is thus a potential therapeutic target for mitigating inflammatory and age-related diseases [15, 16]. It positively regulates conditions such as diabetes, atherosclerosis, and organ damage [16–19]. Current research indicates that SIRT1 can modulate markers associated with oxidative stress, inflammatory responses, mitochondrial function, autophagy, and apoptosis. Myeloid sirtuin1 deficiency exacerbates hippocampal inflammation in mice fed a high-fat diet [20], promotes insulin resistance and exacerbates inflammatory responses [21]. The regulation of macrophage phenotypes by SIRT1 can impact the quality of wound healing [22]. Moreover, studies on diabetic patients have revealed that SIRT1 is underexpressed in many kinds of human tissues, such as the serum and myocardium [23, 24], indicating that SIRT1 protects against diabetes-related diseases. Therefore, elucidating the mechanisms by which SIRT1 participates in regulating macrophage polarization under diabetic conditions and exploring effective intervention strategies are important.

Regenerative therapy, especially stem cell therapies, has immense potential in regenerative repair [25, 26], exhibit unique properties such as multilineage differentiation capability and self-renewal. Furthermore, they are integral to modulating immune responses, suppressing inflammation, secreting various growth factors, and facilitating tissue regeneration processes [27–29]. MSCs primarily function through paracrine mechanisms, where exosomes secreted by these cells act as key mediators of their therapeutic functions [30]. These nano-sized extracellular vesicles, naturally released by cells, serve as essential mediators of intercellular signaling. Notably, exosomes originating from MSCs have gained prominence due to their dual capacity to promote tissue regeneration and regulate immune responses [31]. Adipose-derived mesenchymal stem cells (ADSCs) are considered an ideal choice for regenerative therapy owing to their abundant source and ease of acquisition. Although previous studies suggest that ASDC-derived exosomes (ADSC-exos) have the potential to regulate macrophage polarization and promote wound healing [32, 33], the exact mechanisms by which ADSC-exos modulate macrophages to enhance chronic wound healing remain unclear. Here, we demonstrate that high-glucose conditions cause mitochondrial dysfunction in macrophages, leading to increased ROS production, which is a significant factor in M1 macrophage polarization and persistent inflammation in wounds. ADSC-exos can ameliorate macrophage mitochondrial dysfunction, inhibit M1 macrophage polarization, and promote diabetic wound repair. By constructing a myeloid-specific SIRT1 knockout mouse model of diabetic wounds and exploring the underlying regulatory mechanisms, we found that ASC exosomes can restore autophagy via sirt1, enhance mitochondrial function, mitigate diabetes-mediated macrophage mitochondrial dysfunction, suppress oxidative stress, and promote the reprogramming of macrophages toward M2 polarization, thereby improving the inflammatory environment of wounds and facilitating diabetic wound healing.

## Methods

### Ethics statement

The animal study was carried out following the principles of the ARRIVE guidelines. The research protocol was approved by the Ethics Committee of Xijing Hospital, which is affiliated with the Air Force Medical University. Human tissues were obtained from those patients who needed surgery for treatment. Written contents were acquired from patients or their legal guardians.

### Isolation, culture, and identification of ADSCs

Normal human subcutaneous adipose tissue was obtained from young female patients who underwent liposuction at the Department of Plastic surgery, Xijing Hospital (Xi’an, China). All donors provided written informed consent after receiving comprehensive information regarding tissue utilization. Following extraction, the tissues underwent enzymatic digestion using 1 mg/mL collagenase type I (Gibco BRL, USA) with continuous agitation at 37°C for 60 min. Post-digestion processing included sequential filtration through 100 μm and 70 μm meshes followed by centrifugation (200 × g, 5 min). The pelleted cells were subsequently resuspended in DMEM supplemented with 10% fetal bovine serum (Gibco BRL, USA), and plated at 3.0 × 10^4^/cm^2^ density for culture under standard conditions (37°C, 5% CO₂). Flow cytometry (BD, USA) was used to detecting CD31, CD34, CD90, and CD105 expression. To assess multipotent differentiation capacity, ADSCs were subjected to adipogenic induction for 14 days and osteogenic induction for 21 days. Then cells were fixed with 4% paraformaldehyde and stained with oil red O or alizarin red S following the manufacturer’s instructions to detect the results of the induction culture. Images were viewed under an FSX100 microscope (Olympus, Tokyo, Japan).

### Isolation and characterization of ADSC-derived exosomes (ADSC-exos)

ADSCs from passages 3–5 were utilized for further experiments. When the cells reached 80% confluence, the culture medium was transitioned to DMEM-F12 medium supplemented with 10% exosome-depleted serum. Twenty-four hours later, cellular supernatants were collected to extract the exosomes via the differential ultrahigh-speed centrifugation method. Morphological character of exosomes was detected by transmission electron microscopy (TEM). To assess the expression of the exosome-specific markers CD9 and CD63, western blotting was used. ADSC-exos were stored at −80°C.

### Cell culture, grouping and interventions

RAW264.7 cells were were acquired from the American Type Culture Collection (ATCC, USA)，while primary peritoneal macrophages were isolated from C57BL/6 J mice. All cells were maintained in RPMI 1640 medium (Gibco, USA) supplemented with 10% fetal bovine serum and 1% penicillin/streptomycin under standard culture conditions (37°C, 5% CO₂). For experimental procedures, RAW264.7 cells were plated in 6-well culture dishes (1 × 10^5^ cells/well). In the high-glucose group, the cells were exposed to 30 mM glucose. To maintain osmotic balance, the control cells were exposed to 30 mM D-mannitol. The concentration of ADSC-exos was 1.25 × 10^9^ particles/ml. Following 72-hour treatment protocols, cellular components and conditioned media were systematically collected for downstream analyses.

### Animal model and grouping

Adult male C57 mice that were healthy and of clean grade (with a weight ranging from 25 to 30 g), as well as myeloid-specific *sirt1* knockout mice on a C57 background (*sirt1^−/−^* mice), were acquired from the Center of Experimental Animals of Air Force Military Medical University (Xi’an, China). The Animal Experiments Ethics Committee of Xijing Hospital carried out all the experimental protocols. Following 8 weeks of high-fat feeding, low doses of streptozotocin (STZ) (25 mg/kg) were administered intraperitoneally for 2 consecutive days to induce a diabetic mouse model. All the mice exhibited fasting blood glucose levels>16.7 mmol/L. Some of the mice were sacrificed to obtain peritoneal macrophages and serum. Two weeks after STZ administration, the mice were randomly assigned to different groups. All surgical procedures w ere carried out in a sterile environment under the inhalation of 2% isoflurane. At the center of each mouse's back, a single, round, full thickness skin wound with a diameter of 10 mm was made. ADSC-exos (25 μl at each point, four points, 1 × 10^10^ particles/ml) or an equal volume of PBS were subcutaneously injected 7 times every other day (Figure 2A). The day of surgery was labeled Day 0. On Days 0, 3, 7, 11, and 14, the injured area was measured in size and photographed. After the wound tissues were collected, the mice were sacrificed. The acquired tissues were stored at −80°C. Wound healing rate (%) = (wound area on Day X)/(wound area on Day 0) × 100%.

### Real-time quantitative polymerase chain reaction (qRT–PCR)

Total RNA was isolated via TRIzol reagent (TaKaRa, Japan), followed by cDNA synthesis with a PrimeScript™ RT reagent kit (TaKaRa, Japan). PCR amplification was conducted on a Bio-Rad CFX96 system employing SYBR Premix Ex Taq II (Takara, Japan) in 20 μL reaction volumes. Gene expression quantification was normalized to GAPDH and calculated using the 2^−ΔΔ^CT method. The PCR mixture was amplified under the following conditions: 95°C for 30 s, followed by 40 circles of 95 °C for 30 s denaturation, 10 s annealing at 60°C, and 15 s elongation at 72°C. Sequence-specific primers were detailed in Table 1.

**Table 1:** Primer sequences used for real-time-PCR analysis

RNA	forward primer	reverse primer
IL-6	5’-GGGACTGATGCTGGTGACAA-3′	5’-TCCACGATTTCCCAGAGAACA-3′
TNF-α	5’-GAACTGGCAGAAGAGGCACT-3’	5’-CATAGAACTGATGAGAGGGAGG-3’
MCP-1	5′- GTTAACGCCCCACTCACCTG-3’	5’-CCCATTCCTTCTTGGGGTCA-3’
Arg1	5’-CTCCAAGCCAAAGTCCTTAGAG-3’	5’-AGGAGCTGTCATTAGGGACATC-3’
iNOS	5’-CCAAGCCCTCACCTACTTCC-3’	5’-CTCTGAGGGCTGACACAAGG-3’
IL-1β	5′- TCCAGGATGAGGACATGAGCAC-3’	5′- GAACGTCACACACCAGCAGGTTA-3’
CD206	5’-AAACACAGACTGACCCTTCCC-3’	5’-GTTAGTGTACCGCACCCTCC-3’
GAPDH	5’-GTGTTCCTACCCCCAATGTG-3’	5’-CATCGAAGGTGGAAGAGTGG-3’

### Western blotting

Protein lysates (40 μg/sample) were resolved by sodium dodecyl sulfate and polyacrylamide gels and electrophoretically transferred to nitrocellulose membranes, which were blocked with 5% nonfat milk for 3 h and incubated with primary antibody (1:1000) overnight at 4°C. Primary antibodies against CD9, CD63, MCP-1, IL-1β, iNOS, Arg-1, CD206, TSG101, MFN2, Drp-1, PCG-1α, p-AMPK, AMPK, BECN1, ATG5, LC3II/I, P62, TFEB, CTSB, LAMP1 SIRT1 and β-actin were purchased from Abacm, UK. After washing three times, horseradish peroxidase (HRP)-conjugated secondary antibody (1:3000, Boster, Wuhan) was added, the samples were incubated at room temperature for 1 h. Signal detection was performed using enhanced chemiluminescence (ECL, Millipore, USA) on a FluorChem FC imaging system (Alpha Innotech). Quantitative normalization was achieved through β-actin reference.

### ATP content detection

Following anesthesia induction, murine peritoneal macrophages were isolated and plated in culture dishes. After 2-hour DMEM incubation, adherent cells were washed with PBS to remove non-adherent components. The purified macrophage population was then seeded in 6-well plates (1 × 10^5^ cells/mL) and lysed according to manufacturer specifications (Abcam, UK).

Lysates underwent centrifugation (13 000 × g, 5 min, 4°C) to obtain clarified supernatants. For colorimetric analysis, 50 μL aliquots were loaded in triplicate into 96-well plates. After 30-minute dark incubation at room temperature, absorbance was measured at 570 nm wavelength. ATP concentrations were normalized to total protein content and expressed as nmol/mg protein.

### Hematoxylin and eosin (H&E) staining

Mouse skin tissues from the border of the wounds were acquired and fixed with 4% paraformaldehyde, dried, embedded, sliced, and stained with hematoxylin and eosin. The slices were examined under an FSX100 microscope (Olympus, Tokyo, Japan).

### Immunohistochemical and immunofluorescence staining

For immunohistochemical staining, the wound margin tissue of the mice was sliced and blocked with 5% BSA (1 h, room temperature). Primary antibodies, including those against IL-1β (1:200, Abcam, UK), IL-6 (1:200, Abcam, UK), TNF-α (1:200, Abcam, UK), SIRT1 (1:100, Abcam, UK) and F4/80 (1:200, Abcam, UK), were added separately to the sections in a wet box (16 h, 4°C). After being washed with PBS, sections were incubated with HRP-conjugated secondary antibodies (1:200; Abcam, UK) for 30 minutes at room temperature prior to DAB chromogenic development.

For F4/80 staining, tissue slides were stained with an anti-F4/80 monoclonal antibody (1:100, Abcam, UK) according to the manufacturer’s instructions.

For immunofluorescence staining, the tissues were sliced and blocked in PBS containing 5% bovine serum albumin and primary antibodies (2 h, room temperature), including those against iNOS (1:200, Abcam, UK) and Arg1 (1:200, Abcam, UK). After being washed with PBS, the slides were incubated with fluorescence-conjugated secondary antibodies (Alexa Fluor, Invitrogen) in blocking solution for 1 h and counterstained with DAPI.

All microscopic imaging was performed using an FSX100 system (Olympus, Japan) with matched exposure parameters.

### Determination of ROS levels in cells by flow cytometry

The macrophages were divided into different groups according to the experimental requirements. A ROS detection kit (Chemstan, Wuhan, China) was used to detect the ROS levels in the cells of each group. Specifically, the cells were collected and rinsed with PBS, stained for 20 min in the dark, and then rinsed with PBS 3 times. FlowJo software was used for the analysis of the flow cytometry data.

### JC-1 staining and mitochondrial staining in cells with MitoTracker

Mitochondrial membrane potential (ΔΨm) assessment was performed using JC-1 fluorescent probe (Beyotime, China). Pretreated macrophages were washed three times with PBS, and then, 1 ml of JC-1 working solution was added to each sample (20 min, 37°C). Then macrophages were washed twice with JC-1 staining buffer, and microscopic imaging was performed using an FSX100 system (Olympus, Japan).

For mitochondrial mass visualization, RAW264.7 cells were labeled with MitoTracker Red CMXRos (Beyotime Biotechnology, China) following standard protocols.

### RNA interference-mediated gene silencing

For SIRT1 knockdown studies, RAW264.7 cells at 60–70% confluency were subjected to a serum-depleted medium for 12 h and then were transfected with SIRT1 siRNA(Shanghai Hansheng Technology, China). The negative controls were transfected with LipofiterTM (Invitrogen, USA) according to the manufacturer's instructions.

### Elisa

The serum levels of MCP-1 and IL-1β of mice were assessed via commercial ELISA kits according to the manufacturer's instructions (Cusabio Biotech, Wuhan, China).

### Statistical analysis

Statistical analysis was performed using SPSS 26.0 software (IBM, USA), with results expressed as mean ± standard deviation. For comparisons involving two groups, Student’s t-test was applied, while multigroup comparisons were conducted using analysis of variance (ANOVA). Post hoc analysis was carried out with the LSD test. In cases involving two factors, two-way ANOVA was employed. A significance level of P < 0.05 was adopted for all statistical evaluations.

## Results

### Identification of ADSCs and ADSC-exos

**Figure 1:**
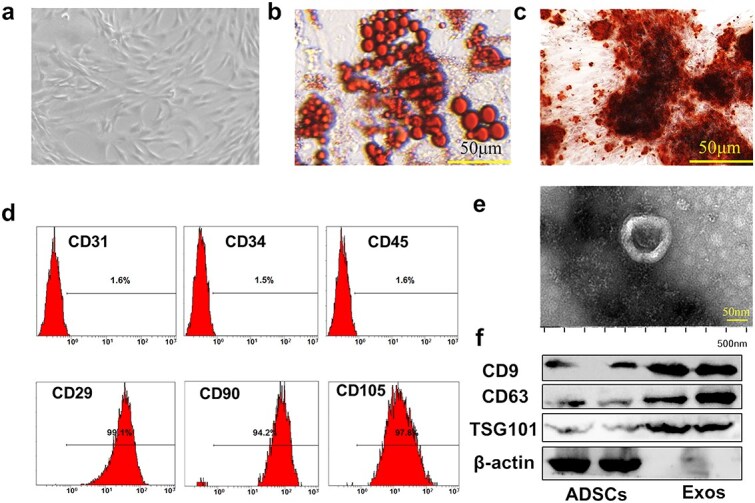
Identification of ADSCs and ADSC-exos. (**a**) ADSCs morphology under microscope. (**b**-**c**) Oil red O and alizarin red S staining were used to investigate adipogenic and osteogenic differentiation, respectively (scale bar = 50 μm). (**d**) Flow cytometry analysis revealed that CD31, CD34, and CD45 were expressed negatively (1.6%, 1.5%, and 1.6%, respectively), whereas CD29, CD90, and CD105 were expressed positively (99.1%, 94.2% and 97.8%, respectively). (**e**) TEM analysis of ADSC-exos (scale bar =50 nm; the length of the figure is 500 nm). (**f**) Western blot analysis of CD9, CD63, and TSG101 in ADSC-exos. *ADSC* adipose mesenchymal stem cell, *ADSC-exos/exos* adipose mesenchymal stem cell-derived exosomes, TEM transmission electron microscopy

ADSCs isolated from adipose tissue exhibited a typical spindle shape (Figure 1a). After 12 days of induction, Oil Red O and Alizarin Red staining demonstrated the multilineage differentiation potential of ADSCs (Figure 1b-c). The ADSC surface markers CD29, CD90, and CD105 showed positive expression, whereas CD31, CD34, and CD45 did not, suggesting the successful isolation of ADSCs (Figure 1d). ADSC-exos were were isolated through differential centrifugation, and revealed a typical cup-shaped structure under TEM (Figure 1e). Western blot analysis further confirmed the presence of the exosomal surface proteins CD63, CD9, and TSG101 (Figure 1f). These results suggested that ADSCs and ADSC-exos were isolated successfully.

### ADSC-exos are capable of reducing the inflammatory response in diabetic wounds and inhibiting macrophage polarization toward the M1 phenotype

**Figure 2:**
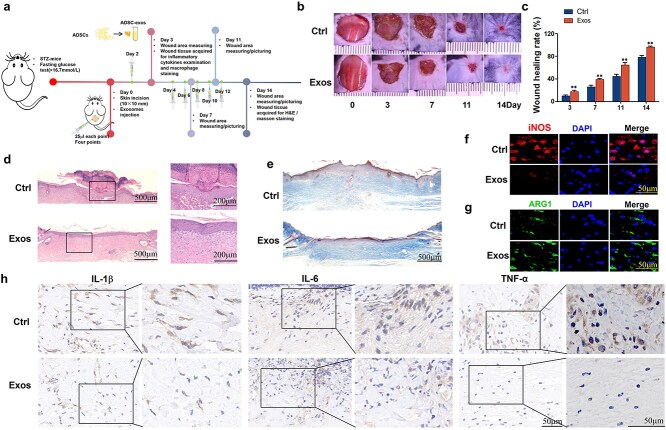
Effects of ADSC-exos on wound healing in STZ-induced diabetic mice. (**a**) Illustration of the experimental process. (**b**) Images of PBS- or ADSC-exo-treated wounds on days 0, 3, 7, 11, and 14 after surgery (n = 3). (**c**) Healing rates of the two groups of mice within 14 days (n = 6). (**d**) H&E staining of wound tissue from control group mice and ADSC-exos group mice on day 14 (scale bar = 500 μm, n = 3). (**e**) Masson staining of wound tissue from control group mice and ADSC-exos group mice on day 14 (scale bar = 500 μm, n = 3). (**f**-**g**) Immunofluorescence staining of iNOS and Arg1 in wound tissue 3 days after surgery in the two groups of mice (scale bar = 50 μm). (**h**) Immunohistological staining of IL-1β, IL-6, and TNF-α in the margin tissue of wounds from the two groups of mice 3 days after surgery (scale bar = 50 μm). The results are presented as the mean ± standard deviation (^*^p < 0.05, ^*^^*^p < 0.01). *STZ* streptozotocin, *Ctrl* control, *ADSC* adipose mesenchymal stem cell, *ADSC-exos/exos* adipose mesenchymal stem cell-derived exosomes, *H&E* Hematoxylin–eosin

100 μl of PBS or 100 μl (25 μl at each point, four points, 1 × 10^10^ particles/ml) of exosomes were injected subcutaneously around the incision 7 times (Figure 2a). The wound areas were recorded. The wound area in the ADSC-exos group was significantly smaller than that in the control group from Day 3 to Day 14. By Day 14, the wounds in the ADSC-exos group had nearly completely healed; in contrast, those in the control group exhibited a significantly longer healing period. H&E staining revealed greater re-epithelialization in the ADSC-exos group than in the control group on Day 14 (Figure 2b-d). On Day 14, wound tissue from the control group and ADSC-exos group was also obtained for Masson staining to assess collagen deposition. Masson staining revealed increased collagen density in the ADSC-exos group compared with the control group (Figure 2e). Immunofluorescence staining of wound tissue (Day 3) revealed a marked reduction in the level of iNOS, an M1 macrophage indicator, in the ADSC-exos group, accompanied by a concomitant increase in the level of Arg1, an M2 macrophage indicator (Figure 2f-g). F4/80 staining also revealed a decrease in the number of macrophages in the tissues of the ADSC-exos group (Supplementary Figure S1a). Immunohistological analysis further confirmed a reduction in the levels of the proinflammatory cytokines IL-1β, IL-6, and TNF-α in the tissues of the ADSC-exos group (Figure 2H). These results indicate that ADSC-exos can shorten the wound healing time, reduce macrophage infiltration, inhibit macrophage polarization toward the M1 phenotype, and reduce the level of inflammatory cytokines.

### Elevated glucose impairs mitochondrial function and promotes M1 polarization, which increases inflammation both *in vivo* and *in vitro*

**Figure 3:**
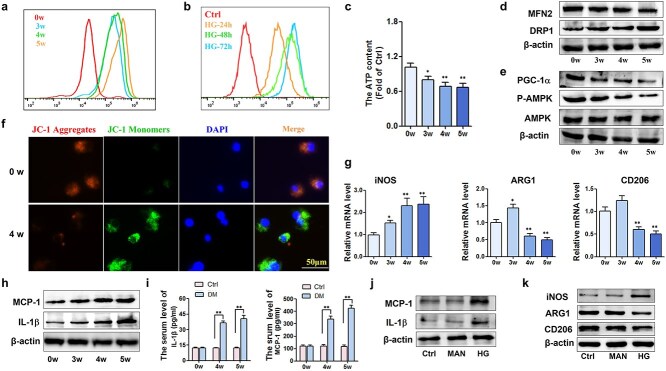
High levels of glucose led to increased ROS and impaired mitochondrial function, which induced a macrophage phenotype shift and inflammatory cytokine expression *in vivo* and *in vitro*. High-fat diet-fed mice were given STZ intraperitoneally for 2 consecutive days, and high glucose-stimulated RAW264.7 cells were used. At 0, 3, 4, and 5 weeks after the use of STZ, peritoneal macrophages and serum were collected for testing. (**a**) ROS levels in macrophages from different periods of STZ-induced mice were measured via flow cytometry. (**b**) ROS levels in RAW264.7 cells treated with high glucose were measured via flow cytometry. (**c**) ATP content of peritoneal macrophages from different periods of STZ induction determined with an ATP test kit. (**d**) Mitochondrial function indicators (MFN2 and Drp-1) were detected by western blotting at different time points. (**e**) Mitochondrial genetic indicators (PCG-1α and *p-*AMPK) were detected via western blotting. (**f**) JC-1 signals of the mitochondrial membrane potential (mtmP) in macrophages from different periods of STZ induction were detected via fluorescence confocal microscopy (scale bar = 50 μm). (**g**) At weeks 0, 3, 4, and 5, peritoneal macrophages from the STZ-induced mice were isolated and examined via qRT–PCR to determine their iNOS, Arg-1, and CD206 mRNA levels. (**h**-**i**) Serum levels of MCP-1 and IL-1β were measured in high-fat diet-fed mice with or without STZ injection at weeks 0, 4, and 5. (**j**) Western blot results showing the protein levels of MCP-1 and IL-1β in the control group, mannitol group (30 mM), and high-glucose group (30 mM) after 72 h of culture. (**k**) The protein levels of iNOS, Arg-1, and CD206 were detected by western blotting in RAW264.7 cells in the control group, mannitol group (30 mM), and high-glucose group (30 mM) after 72 h of culture. The results are presented as the means ± standard deviations (^*^ p < 0.05, ^*^^*^ p < 0.01). n = 3; *Ctrl* control, *MAN* mannitol, *HG* high glucose, *STZ* streptozotocin

Peritoneal macrophages were isolated from STZ-induced diabetic mice at different time points (0, 3, 4, and 5 weeks postinduction). RAW264.7 cells were treated with high glucose as described previously. Flow cytometry was used to quantify reactive oxygen species (ROS) levels in macrophages. In a high-glucose environment, the levels of ROS increased significantly both *in vivo* and *in vitro* (Figure 3a-b). The ATP content in peritoneal macrophages was notably diminished following STZ treatment (Figure 3c). The active mitochondria in those cells were stained with MitoTracker (Supplementary Figure S1b). These observations collectively indicated compromised mitochondrial function, manifested by elevated ROS levels and reduced ATP synthesis. To investigate the status of mitochondrial homeostasis, which involves mechanisms such as fission, fusion, mitophagy, and biogenesis [34], western blotting was conducted to assess the protein levels of the mitochondrial activity markers MFN2 and Drp-1, as well as the mitochondrial biogenesis markers PGC-1α and phosphorylated AMPK. In the context of diabetes, a clear pattern emerged in which MFN2, PGC-1α, and phosphorylated AMPK levels were reduced, whereas Drp-1 levels were elevated (Figure 3d-e, Supplementary Figure S1 c-d). Fluorescence confocal microscopy was used to detect JC-1 signals indicative of the ΔΨm across different time points (Figure 3f, Supplementary Figure S1e). The reduction in MFN2 levels implied a diminished capacity for glucose oxidation, whereas the increase in Drp-1 suggested exacerbated mitochondrial fission. Additionally, reduced AMPK phosphorylation and lower PGC-1α levels are indicative of mitochondrial dysfunction. Collectively, these findings underscore the impaired mitochondrial function exhibited by peritoneal macrophages in diabetic mice.

RT–PCR was used to measure the mRNA levels of iNOS, Arg-1, and CD206. The administration of STZ led to a progressive shift toward the M1 phenotype in peritoneal macrophages, characterized by an increase in iNOS levels and a decrease in Arg-1 and CD206 levels, as depicted in Figure 3g. At 4 and 5 weeks of STZ stimulation, protein levels of MCP-1 and IL-1β in both macrophages and serum were increased significantly (Figure 3h-i). RAW264.7 cells were incubated with 30 mM glucose or mannitol for 72 hours, resulting in a significant increase in MCP-1 and IL-1β protein levels upon glucose treatment. It also induced an increase in iNOS expression and a decrease in the levels of Arg-1 and CD206 (Figure 3j-k, Supplementary Figure S1g-h). Collectively, both *in vivo* and *in vitro* studies demonstrated that elevated glucose levels are capable of inducing inflammation and promoting macrophage polarization toward the M1 phenotype, during which mitochondrial dysfunction was evident.

### ADSC-exos enhanced the mitochondrial function of high glucose–stimulated RAW264.7 cells, reduced ROS production, and inhibited M1 polarization *in vitro*

**Figure 4:**
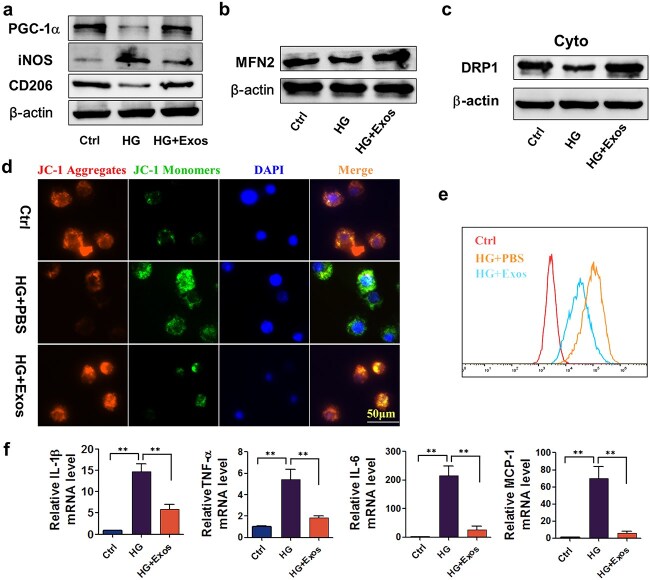
ADSC-exos facilitated the expression of SIRT1 and protected against high glucose-induced mitochondrial dysfunction and M1 polarization in high glucose-induced RAW264.7 cells. (**a**) The protein levels of mitochondrial genetic indicators (PCG-1α) and macrophage polarization markers (iNOS and CD206) were detected by western blotting in each group. (**b**-**c**) MFN levels in macrophages and Drp-1 levels in the cytoplasm were detected separately. (**d**) The mitochondrial membrane potential (mtmP) in each group of macrophages was detected with a JC-1 fluorescent probe. (**e**) ROS levels in the macrophages in each group were measured via flow cytometry. (**f**) The mRNA levels of IL-1β, TNF-α, IL-6, and MCP-1 in each group of macrophages. Con: Control group, treated with mannitol; HG: High glucose group, treated with high glucose (30 mM); HG + EXO: Treated with high glucose (30 mM) and ADSC-exos (1.25 × 10^9^ particles/ml). The results are presented as the means ± standard deviations (n = 3, ^*^p < 0.05, ^*^^*^p < 0.01). *Ctrl* control, *HG* high glucose, *ADSC-exos/exos* adipose mesenchymal stem cell-derived exosomes

To explore the impact of ADSC-exos on mitochondrial function and macrophage polarization under high-glucose conditions in greater detail, RAW264.7 cells stimulated with high glucose were used. Compared with those in the control group, macrophages exposed to high glucose presented markedly decreased levels of PCG-1α and CD206 and increased iNOS. However, pretreatment with ADSC-exos partially mitigated these changes (Figure 4a, Supplementary Figure S2a). Subsequently, western blotting was conducted to assess the protein levels of MFN2 in macrophages and Drp-1 in the cytoplasmic fraction. Stimulation with glucose resulted in a decrease in MFN2 levels, which were conversely elevated upon ADSC-exos treatment. Similarly, high-glucose stimulation led to a reduction in cytoplasmic Drp-1, whereas ADSC-exo administration elicited a substantial increase in Drp-1 levels (Figure 4b-c, Supplementary Figure S2 b-c). A previous study indicated that a deficiency in Drp-1 impairs autophagy in hepatocytes [35]. The data revealed that pretreatment with ADSC-exos ameliorated the compromised mitochondrial function caused by high glucose. Additionally, the application of ADSC-exos to RAW264.7 cells partially reversed the decrease in the mitochondrial membrane potential caused by high glucose and decreased the ROS level (Figure 4d-e, Supplementary Figure S2 d-e), which indicated that high glucose led to significantly decreased ATP in the cells. The active mitochondria in RAW264.7 cells were stained with MitoTracker, which also revealed impaired mitochondria in a high-glucose environment. Moreover, with the use of ADSC-exos, the fluorescence intensity increased (Supplementary Figure S2f). Furthermore, ADSC-exos effectively suppressed the levels of proinflammatory cytokines, including IL-1β, TNF-α, IL-6, and MCP-1, compared with those in cells treated with high glucose alone (Figure 4f). These findings unequivocally demonstrate that the administration of ADSC-exos offers protection against mitochondrial dysfunction, thereby preventing ROS buildup and sustaining inflammation.

### Both *in vitro* and *in vivo*, ADSC-exos were shown to promote autophagy flux in macrophages and augment mitochondrial activity

**Figure 5:**
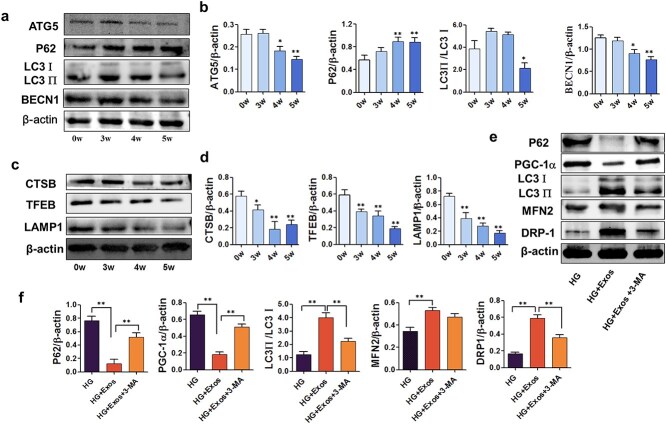
The autophagy–lysosomal pathway in macrophages was impaired in STZ-induced diabetic mouse peritoneal macrophages, and the use of ADSC-exos improved high glucose-induced impairment by regulating autophagy. (**a**-**d**) Peritoneal macrophage protein levels of BECN1, ATG5, LC3II/I, P62, TFEB, CTSB, and LAMP1 were detected via western blotting at 0, 3, 4, and 5 weeks after the use of STZ. (**e**-**f**) The protein levels of LC3II, P62, ATG5, PCG-1α, MFN2, and Drp-1 in different groups of RAW264.7 cells were detected via western blotting. HG: High glucose group treated with high glucose (30 mM); HG + EXO: Treated with high glucose (30 mM) and ADSC-exos (1.25 × 10^9^ particles/ml); HG + EXO + 3-MA: Treated with high glucose (30 mM), ADSC-exos (1.25 × 10^9^ particles/ml) and 3-MA (10 mM). The results are presented as the means ± standard deviations (n = 3, ^*^p < 0.05, ^*^^*^p < 0.01). *3-MA* 3 methyladenine, *Ctrl* control, *HG* high glucose, ADSC-exos/exos Adipose mesenchymal stem cell-derived exosomes

Peritoneal macrophages were harvested from STZ-induced diabetic mice at various time points (0, 3, 4, and 5 weeks postinduction). Western blotting was used to measure the protein levels of autophagy and lysosome markers, including beclin 1 (BECN1), autophagy-related 5 (ATG5), LC3II, LC3I, P62, transcription factor EB (TFEB), cathepsin B (CTSB), and lysosomal-associated membrane protein 1 (LAMP1) (Figure 5a-d). *In vitro*, the autophagy-related indices were impaired. Intriguingly, all the aforementioned proteins were significantly altered 4 weeks after STZ administration, with shifts in CTSB, TFEB, and LAMP2 manifesting earlier. These findings collectively point to compromised autophagy flux and lysosomal functionality. Furthermore, lysosomal dysfunction occurs earlier than autophagic flux does. LC3 II/I slightly increased 3 weeks after the use of STZ and decreased later. However, the changes were not statistically significant. RAW264.7 cells were divided into the control group, HG group, HG + Exos group, and HG + Exos+3-MA (10 mM, Sigma–Aldrich, US) groups. In a high-glucose environment, with the use of ADSC-exos, mitochondrial function was partially rescued, since the levels of P62 and PGC-1α decreased, whereas the levels of LC3 II/I, MFN2, and Drp1 increased. It was intriguing to investigate whether the enhancement of macrophage mitochondrial function was associated with autophagic flux. The administration of 3-methyladenine (3-MA), an autophagy inhibitor, partially reduced the effects of ADSC-exos (Figure 5e-f). These results indicate that ADSC-exos can improve autophagy flux to salvage mitochondrial function.

### SIRT1 is key for the ability of ADSC-exos to improve mitophagy in diabetic wounds

**Figure 6:**
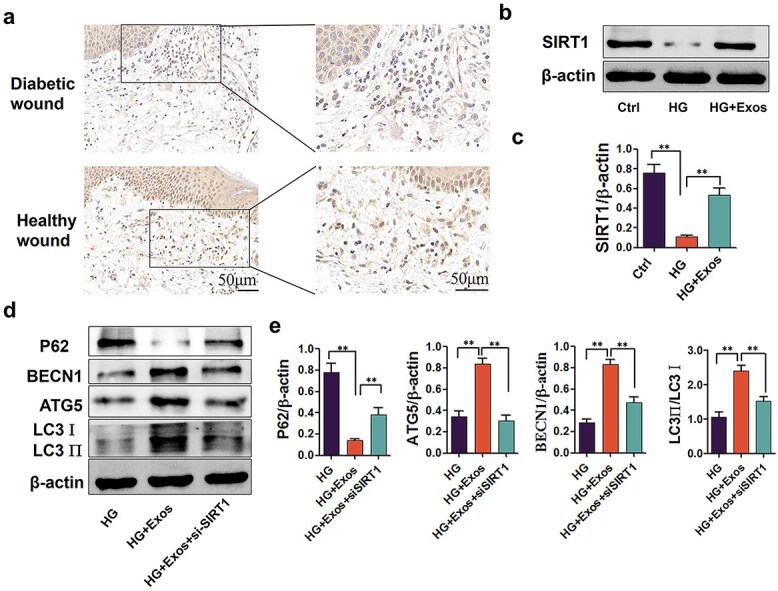
SIRT1 was suppressed in diabetic wound tissue and in high glucose-cultivated macrophages. Suppression of SIRT1 diminished the effect of ADSC-exos *in vitro*. (**a**) Immunohistological staining of SIRT1 in wound margin tissues from diabetic foot wounds or acute wounds from patients without diabetes (n = 6). (**b**-**c**) Protein levels of SIRT1 in each group of RAW264.7 cells. (**d**-**e**) The protein levels of BECN1, P62, ATG5, and LC3-II in RAW264.7 cells were detected by western blotting. HG: High glucose group treated with high glucose (30 mM); HG + EXO + NC: Treated with high glucose (30 mM) and ADSC-exos (1.25 × 10^9^ particles/ml) + negative control siRNA; HG + EXO + NC: Treated with high glucose (30 mM) and ADSC-exos (1.25 × 10^9^ particles/ml) + SIRT1 siRNA. The results are presented as the means ± standard deviations (n = 3, ^*^p < 0.05, ^*^^*^p < 0.01). *Ctrl* control, *HG* high glucose, *ADSC-exos/exos* adipose mesenchymal stem cell-derived exosomes

Diabetic wound tissues were collected from patients suffering from diabetic foot ulcers requiring debridement. Healthy wound tissues were acquired from nondiabetic adults who had experienced traumatic injuries within five days. SIRT1 levels in the wound border tissues were assessed. SIRT1 expression in diabetic tissue was lower than that in healthy wound tissue (Figure 6a). In high glucose–treated RAW264.7 cells, the level of SIRT1 decreased significantly, while the use of ADSC-exos partially rescued the decrease in the SIRT1 level (Figure 6b-c). In a high-glucose environment, the levels of BECN1, ATG5, and LC3II/I were elevated with the use of ADSC-exos, whereas with the transfection of SIRT1 siRNA, the effect of ADSC-exos was almost diminished (Figure 6d-e). These findings indicate that SIRT1 knockdown reduces autophagic flux and diminishes the protective effects of ADSC-exos.

### SIRT1 deletion diminished the effects of ADSC-exos on macrophages, including improvements in mitochondrial activity and the inhibition of inflammatory responses

**Figure 7:**
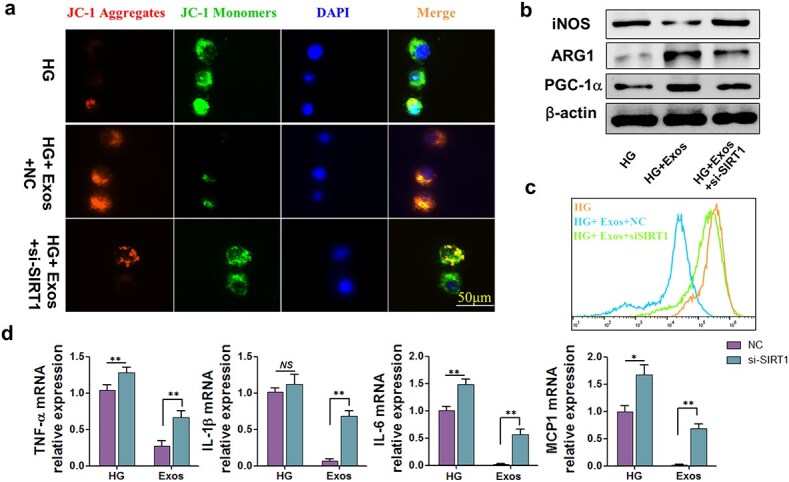
Effects of SIRT1 deletion on mitochondrial function and inflammation in high glucose–cultivated macrophages. (**a**) JC-1 signals of the mitochondrial membrane potential (mtmP) in RAW264.7 cells were measured via fluorescence confocal microscopy (scale bar = 50 μm). (**b**) The protein levels of SIRT1, iNOS, Arg-1, and PGC-1α in RAW264.7 cells were detected by (**c**) ROS levels in RAW264.7 cells were assessed via flow cytometry. (**d**) The mRNA levels of TNF-α, IL-1β, IL-6, and CCL2 in RAW264.7 cells. HG: High glucose group treated with high glucose (30 mM); HG + EXO: Treated with high glucose (30 mM) and ADSC-exos (1.25 × 10^9^ particles/ml) + negative control siRNA; HG + EXO + siSIRT1: Treated with high glucose (30 mM) and ADSC-exos (1.25 × 10^9^ particles/ml) + SIRT1 siRNA. The results are presented as the means ± standard deviations (n = 3; ^*^p < 0.05, ^*^^*^p < 0.01). *HG* high glucose, *ADSC-exos/exos* adipose mesenchymal stem cell-derived exosomes

High-glucose stimulation resulted in instability in maintaining the mitochondrial membrane potential (ΔΨm). Conversely, the use of ADSC-exos restored the ΔΨm of macrophage mitochondria. However, in macrophages transfected with SIRT1 siRNA, the effects of ADSC-Exos were partially diminished (Figure 7a, Supplementary Figure S3a). PGC-1α, a central regulator of mitochondrial biogenesis and function, was also increased with the use of ADSC-exos and decreased with the depletion of SIRT1 (Figure 7b, Supplementary Figure S3b). Macrophages were also polarized to the M1 phenotype with the depletion of SIRT1, as illustrated in Figure 7b, where the levels of Arg-1 decreased while the levels of iNOS increased. The accumulation of ROS in macrophages followed a similar trend (Figure 7c). In a high-glucose environment, the levels of inflammatory cytokines, including TNF-α, IL-1β, IL-6, and CCL2 increased, while the use of ADSC-Exos relieved inflammation. However, with SIRT1 knockdown *in vitro,* the levels of these inflammatory cytokines rebounded (Figure 7d).

### In diabetic mice with SIRT1 deletion, there was a significant increase in macrophage infiltration, which was polarized toward the M1 phenotype. The absence of SIRT1 resulted in delayed wound healing

**Figure 8:**
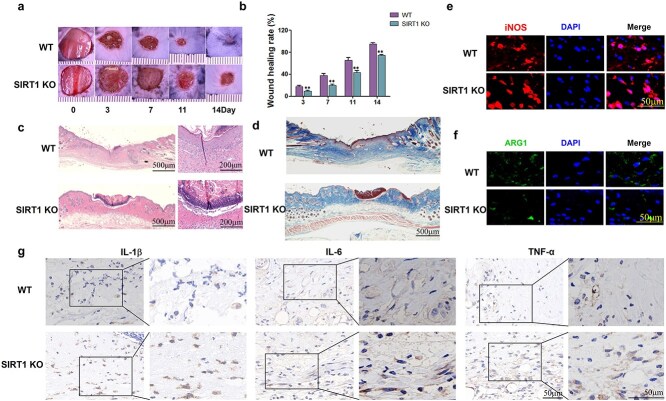
SIRT1 knockout delayed wound healing and increased inflammation in STZ-induced mice. (**a**) Digital images of the wound regions of the mice on days 0, 3, 7, 11, and 14 (scale bar = 1 cm). (**b**) Wound healing rates of the two groups of mice (n = 6). (**c**) H&E staining of wound tissue of two groups mice on day 14 (scale bar = 500 μm, n = 3) (**d**) Masson staining of wound tissue on day 14 (scale = 500 μm). (**e**-**f**) Immunofluorescence staining of iNOS and Arg1 in wound tissue 3 days after surgery in the two groups of mice (scale bar = 50 μm). (**g**) Immunohistology staining of IL-1β, IL-6, and TNF-α in the margin tissue of wounds from the two groups of mice 3 days after surgery (n = 3, scale bar = 50 μm). The results are presented as the means ± standard deviations (^*^p < 0.05, ^*^^*^p < 0.01). *KO* knockout, *WT* wild type, *STZ* streptozotocin

A skin incision model was established in STZ-induced diabetic myeloid-specific *sirt1*^−/−^ mice and littermate wild-type C57BL/6 J mice. On Days 0, 3, 7, 11, and 14, the wound areas were recorded. SIRT1 suppression decreased the wound healing rate (Figure 8a-b) and led to an increase in the infiltration of inflammatory cells (Figure 8c). On Day 14, Masson staining of the wound edge tissues revealed that the collagen sediment in the *sirt1*^−/−^ mice was thinner than that in the control mice (Figure 8d). The M1 marker iNOS was also elevated in the si-SIRT1 group, whereas Arg1 was decreased in the same Group 3 days postsurgery (Figure 8e-f). The levels of inflammatory cytokines, including TNF-α, IL-1β, and IL-6, were also elevated in *sirt1*^−/−^ mouse tissue (Figure 8g). These findings demonstrated that the suppression of SIRT1 aggravated inflammation and promoted macrophage infiltration and polarization to the M1 phenotype.

## Discussion

In diabetic patients, continuous exposure to a hyperglycemic environment impairs immune function and induces various nerve dysfunctions and vascular problems. These factors, in turn, exacerbate lower extremity injuries and lead to diabetic foot ulcers [2]. As the global prevalence of diabetes is on the rise, the demand for the care and treatment of diabetic wounds is also increasing. Delayed wound healing frequently results in amputation, which has a significant impact on patients' quality of life. In recent years, scientists have explored the mechanisms of diabetic wound healing; however, the pathological and physiological processes involved remain unclear, creating difficulties for the prevention and management of diabetic wounds.

Elevated blood glucose prompts the production of reactive oxygen species (ROS) and advanced glycation end products (AGEs), which initiate inflammation and tissue damage [36, 37]. The impaired resolution of inflammation and persistent inflammatory responses are characteristic features of the pathogenesis of diabetic complications. As key functional cells that participate in the inflammatory response within the tissue microenvironment, macrophages play crucial roles in inflammation and tissue regeneration [8]. In diabetes, macrophage dysfunction results in homeostatic imbalances and abnormal phenotypic transitions, ultimately leading to the prolongation of inflammatory reactions and increased tissue damage [6].

Research has indicated that mitochondrial function directly determines the polarization direction of macrophages [38, 39]. Mitochondria, as highly dynamic organelles, regulate their morphology through a balance of fission and fusion processes, adapting to cellular metabolic requirements. Research indicates that in diabetic conditions, elevated glucose levels upregulate Drp1 protein expression and phosphorylation, prompting its movement from the cytoplasm to mitochondria and enhancing mitochondrial fragmentation [40]. Based on these findings, we further demonstrated that high-glucose conditions in diabetes induce mitochondrial fission overproduction of ROS in macrophages, promoting M1 polarization (Figure 3). PGC-1α and phosphorylated AMPK are markers of mitochondrial biogenesis. The PGC 1 family of regulated coactivators, which includes PGC-1α, PGC-1β and PRC, forms a critical regulatory network that controls the transcriptional regulation of mitochondrial biogenesis and respiratory function. AMPK is a guardian of metabolism and mitochondrial homeostasis [41]. The main form of its activation is the phosphorylation of Thr172 on the α subunit. Then, AMPK can phosphorylate PGC-1α at specific serine and threonine residues, which can increase mitochondrial gene expression. As shown in Figure 3, the levels of p-AMPK and PGC-1α gradually decreased in diabetic mice, which indicated impaired mitochondrial biogenesis [42]. Similarly, multiple studies have suggested that in mitochondria, the polarization of M1 macrophages driven by ROS and the subsequent secretion of pro-inflammatory cytokines contribute to diabetic complications [43, 44].

Recent research has revealed that RNA interference of mitochondrial complexes (Ndufs4-RNAi) induces mitochondrial dysfunction and impairs autophagosome degradation by compromising lysosomal activity [10, 45]. This leads to an increase in M1 macrophages and proinflammatory cytokine levels, suggesting that mitochondrial dysfunction compromises lysosomal function and the autophagic process. In this study, we observed impaired autophagic flux and autophagosome maturation in macrophages from diabetic mice, characterized by a decrease in LC3II and lower levels of autophagosome synthesis-related proteins such as BECN1 and ATG5. A previous report indicated that prolonged reactive oxygen species (ROS) overload induced by high glucose resulted in significant mitochondrial damage in macrophages; however, scavenging mitochondrial ROS improved mitochondrial biogenesis and lysosomal function [10]. A moderate rise in ROS levels can act as a signal to trigger autophagy as a compensatory mechanism. However, when ROS levels become excessively high, they can cause significant oxidative harm to intracellular proteins and organelles. Under stress conditions, transcription factor EB (TFEB) translocated to the nucleus, promoting the enlargement of the lysosomal system and facilitating the breakdown of accumulated lysosomal materials [46, 47]. In this study, we found a decrease in TFEB, LAMP1, and CTSB expression in macrophages three weeks after exposure to high glucose, possibly explaining the inhibition of autophagosome maturation in macrophages. However, BECN1-mediated suppression of TFEB activation, through PGC-1α-regulated mitochondrial biogenesis, reduces mitochondrial mass and decreases cell death in cardiac myocytes under stress [48]. These findings suggested that diabetes may impair lysosomal function and autophagic flux in macrophages, thereby promoting M1 polarization and the release of inflammatory cytokines in macrophages.

ADSC-exos are considered promising options for therapeutic regeneration and repair owing to their potent immunomodulatory and antioxidant properties [32, 33]. In this study, we successfully isolated and identified ADSC-exos. Subsequently, we explored their role in diabetic wound healing. The use of ADSC-exos accelerated the wound healing rate in diabetic mice and suppressed wound inflammatory responses. As shown in Figure 6a, macrophage infiltration was much more pronounced in the wounds of diabetic mice. Supplementary Figure S1a showed that the use of ADSC-exos could also reduce the infiltration of macrophages. However, it has been reported that both high glucose and ADSC-exos could enhance the migration of macrophages [49, 50]. In other words, the decrease in the number of macrophages in the tissue was likely not due to decreased migration ability but rather to suppressed inflammation. Furthermore, we focused on mitochondria, important organelles that impact cell functions.

Our findings provided evidence that, under diabetic conditions, the loss of mitochondrial function in macrophages led to reactive oxygen species (ROS) production, which in turn impaired lysosomes and blocked autophagic flux in macrophages. The impaired autophagic flux further promoted macrophage polarization toward the M1 phenotype and generated a sustained chronic inflammatory response. ADSC-exos constituted an effective intervention strategy that significantly alleviated mitochondrial dysfunction induced by high-glucose factors, enhanced lysosomal function in macrophages, promoted autophagic flux, and consequently facilitated macrophage repolarization, improving the inflammatory environment. This findings also partly elucidated the mechanism by which ADSC-exos promotes wound healing in streptozotocin (STZ)-induced diabetic mice via *in vitro* therapy.

Recent studies have indicated that enhancing the biosynthesis of NAD^+^ or reducing its catabolism can contribute to therapeutic outcomes in diabetic cardiomyopathy [51]. Emerging evidence suggests that dysregulation of NAD^+^ metabolism is increasingly recognized as a critical factor in the pathogenesis of various diabetes complications in individuals with diabetes [52]. By restoring NAD^+^ levels and activating sirtuin 1 (SIRT1), insulin sensitivity is improved, and genes related to oxidative stress, inflammatory responses, and circadian rhythms are activated, thereby improving glucose intolerance [18]. SIRT1 is considered a promising therapeutic target for aging-related diseases, as it may alleviate oxidative stress, reduce inflammatory responses, and reverse mitochondrial dysfunction [53]. Research has shown a decrease in SIRT1 expression in several types of human tissue in diabetic patients [23, 24]. Our preliminary findings indicated that SIRT1 deficiency in diabetic wounds is closely associated with delayed wound healing [54]. This may be related to impaired mitochondrial function. Following the disruption of SIRT1 in macrophages, proteins associated with mitochondrial metabolic pathways were significantly enriched. We further employed a myeloid macrophage SIRT1-knockout mouse model to demonstrate that the expression levels of SIRT1 in macrophages are directly related to the quality of wound healing [22].

Exosomes secreted by MSCs encompass a diverse array of bioactive molecules that mediate the therapeutic effects of MSCs. Mechanistically, this study revealed that ADSC-exos can upregulate the expression of SIRT1, which, in turn, facilitates wound healing by enhancing autophagy, enhancing mitochondrial function, and reprogramming macrophages toward the M2 polarization state. This process alleviated inflammation in wounds and promoted the healing of chronic diabetic wounds. This study provides a preliminary exploration of the potential mechanism by which ADSC-exos promote chronic wound healing under high-glucose conditions through SIRT1 activation. However, the specific mechanisms of downstream signaling pathways and the molecular mechanisms by which ADSC-Exos influence SIRT1 activity remain to be further elucidated. Improving our understanding of these areas will contribute significantly to refining the role of ADSC-exos in diabetic wound management and identifying therapeutic targets for diabetes-related diseases. This knowledge could pave the way for innovative treatments and personalized medicine approaches in diabetes care.

## Conclusions

In summary, we revealed that SIRT1 is a crucial mediator by which ADSC-exos improve mitochondrial function and autophagy levels in macrophages at diabetic wound sites. ADSC-exos mediated SIRT1, improved mitochondrial dysfunction and lysosomal function, and promoted macrophage polarization toward the M2 anti-inflammatory phenotype, thus improving diabetic wound healing. This study revealed that in diabetic mice, lysosome dysfunction occurs prior to mitochondrial function, and SIRT1 could be a clue for programming exosomes for clinical use to improve diabetic wounds.

## Abbreviations

3-MA: 3 methyladenine; ACTB/β-actin: actin beta; ADSC-exos: Adipose mesenchymal stem cell-derived exosomes; ATG5: autophagy related 5; BECN1: beclin 1; CTSD: cathepsin D;GAPDH: glyceraldehyde-3-phosphate dehydrogenase; HG: High glucose; LAMP2: lysosomal-associated membrane protein 2; MEF: mouse embryonic fibroblast; NF-κB; m-TOR: mechanistic target of rapamycin kinase; PGC-1α:; ROS: reactive oxygen species; SIRT1: sirtuin 1; SOD: superoxide dismutase; STZ: Streptozotocin TEM: transmission electron microscopy; TFEB: transcription factor EB; WT: wild type

## Ethics approval and consent to participate

The research protocol was proved approved by the Ethics Committee of Xijing Hospital, affiliated with the Air Force Medical University (KY20203232–1). The animal study was carried out in accordance with the principles of ARRIVE guidelines. The human tissues were obtained from patients who need surgery for treatment. Written contents were acquired from patients or their legal guardians.

## Consent for publication

The authors confirm that the work described has not been published in whole or in part. It is not under consideration for publication elsewhere. No conflict of interest exists in the submission of this manuscript, and all authors have approved this manuscript for publication.

## Supplementary Material

supplementary_material_tkaf017

## Data Availability

Data will be made available on request. The corresponding author and first authors could provide the required data.
