# Supplementary material for: Adipose mesenchymal stem cell-derived exosomes rescue mitochondrial function through SIRT1 to improve diabetic wound healing
Source: Burns Trauma. 2025 Apr 17;13:tkaf017. doi: 10.1093/burnst/tkaf017 (PMC12516946; doi:10.1093/burnst/tkaf017)
Supplement: supplementary_material_tkaf017 [file supplementary_material_tkaf017.docx]

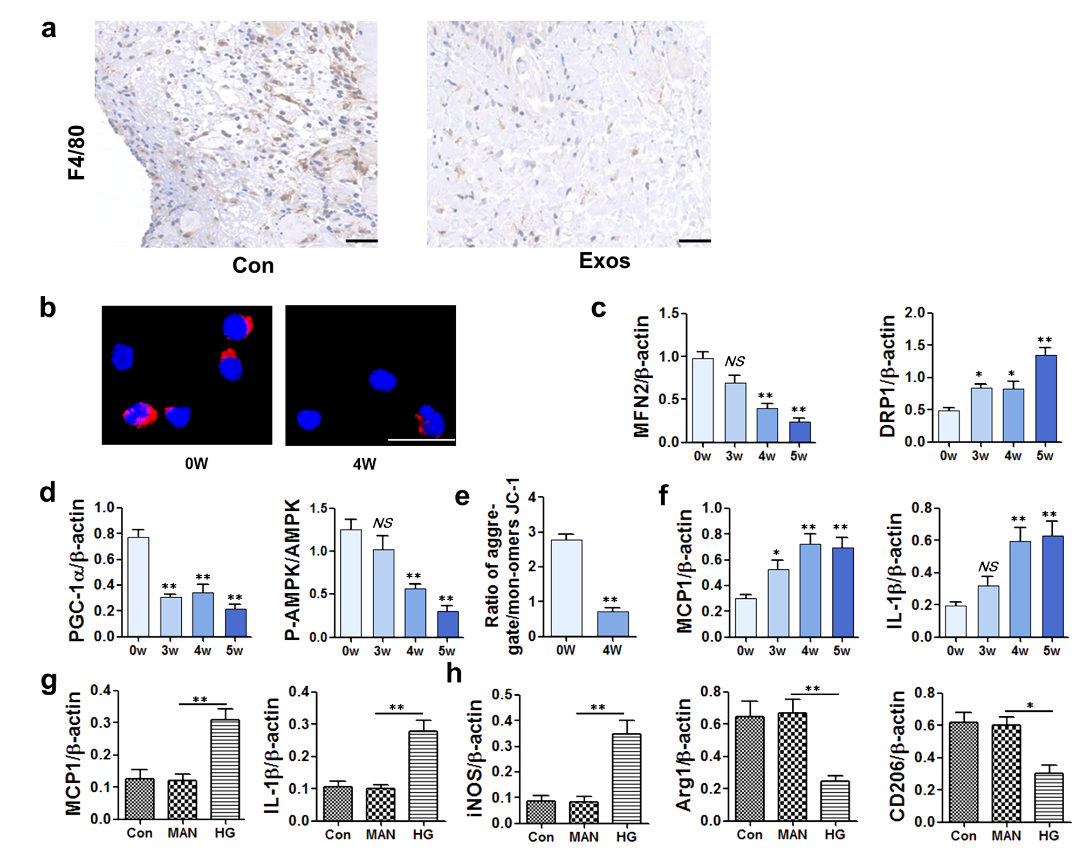


Figure S1 legend. (a). F4/80 staining of wound tissue in mice 14 days after surgery with or without ADSC-exo injection (scale bar = 50 μm). (b) Fluorescence staining showing the localization and activity of mitochondria in peritoneal macrophages at different time point (scale bar = 50 μm). (c-f). Statistical analyze of levels changes of mitochondrial function indicators and inflammation cytokines in mice serum and in peritoneal macrophages in Figure 3. (g-h) Statistical analyze of levels changes inflammatory cytokines and macrophages polarization indicators in Figure 3. The results are presented as the mean ± standard deviation (n=3, * p <0.05, ** p <0.01).


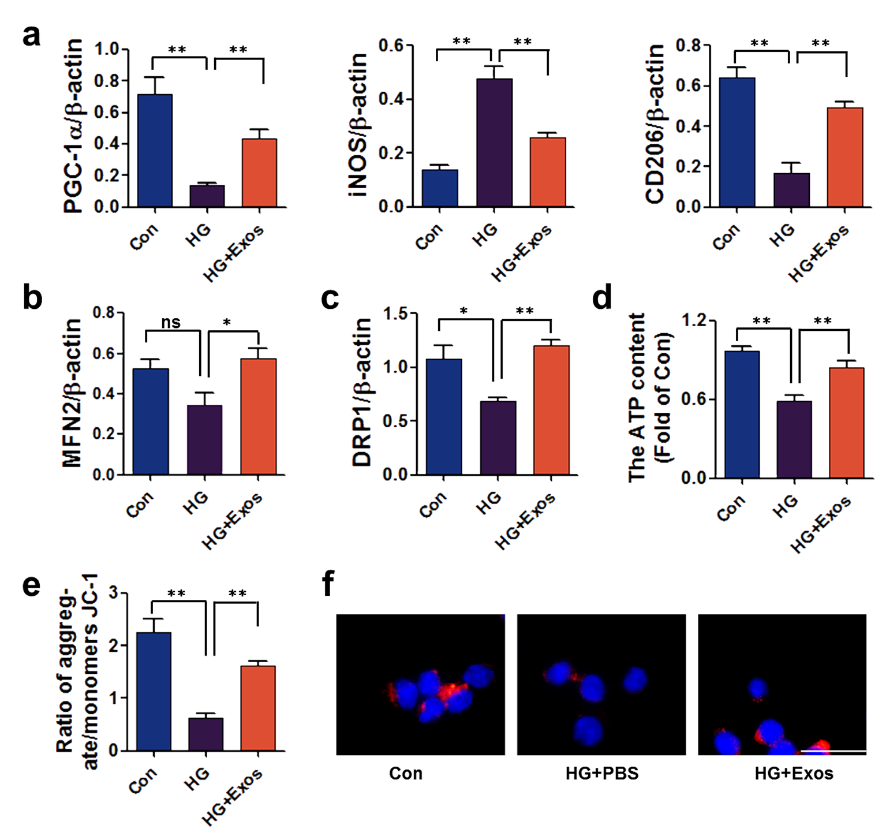


Figure S2 legend. (a-c). The statistical analysis of Figure 4 a-c. They are indicated the differences of mitochondrial function of each group of RAW264.7 cells. (d) ATP content in each group of RAW264.7 cells. (e). Fluorescence staining showing the localization and activity of mitochondria in RAW264.7 cells in each group (scale bar = 50 μm). (f). The statistical analysis of Figure 4d, showing the differences of JC-1 signal fluorescence intensity differences of each group of RAW264.7 cells. The results are presented as the mean ± standard deviation (n=3, * p <0.05, ** p <0.01).


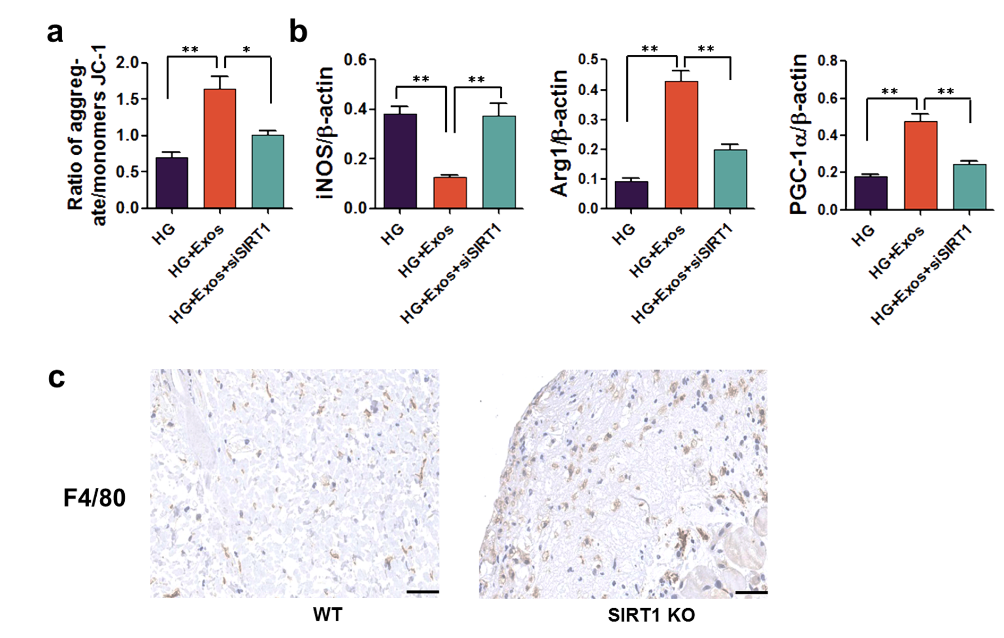


Figure S3 legend. (a). The statistical analysis of Figure 7a, showing the differences of JC-1 signal fluorescence intensity differences of each group of RAW264.7 cells. (b) The statistical analysis of Figure 7B. (c) F4/80 staining of wound tissue in mice 14 days after surgery of WT mice and myeloid specific *sirt1*^-/-^ knockout mice (scale bar = 50 μm). The results are presented as the mean ± standard deviation (n=3, * p <0.05, ** p <0.01).
